# Supplementary material for: Urine dicarboxylic acids change in pre-symptomatic Alzheimer’s disease and reflect loss of energy capacity and hippocampal volume
Source: PLoS One. 2020 Apr 16;15(4):e0231765. doi: 10.1371/journal.pone.0231765 (PMC7162508; doi:10.1371/journal.pone.0231765)
Supplement: S1 Table — P values < 0.05 are shown in bold italics. (DOCX) [file pone.0231765.s001.docx]

S1 Table. Analytical parameters used for detecting and quantifying DCAs in urine samples. Carbon number (C3-C10), negative ion (m/z), retention time (RT), deuterated internal standards, detection linear range, and correlation (R^2^).

| **Name** | **Carbon #** | **m/z** | **RT (min)** | **ISTD** | **Linear Range (ng)**  **LOD Top** | | **R^2^** |
| --- | --- | --- | --- | --- | --- | --- | --- |
| **Malonic acid** | C3 | 283.0 | 6.85 | Succinic acid-d_4_ | 0.587 | 3000 | 0.988 |
| **Succinic acid-d_4_** | C4 | 297.0 | 7.54 | -- | N/A | N/A | N/A |
| **Succinic acid** | C4 | 301.0 | 7.56 | Succinic acid-d_4_ | 0.156 | 750 | 0.971 |
| **Glutaric acid** | C5 | 311.0 | 8.06 | Adipic acid-d_4_ | 0.140 | 750 | 0.974 |
| **Adipic acid-d_4_** | C6 | 329.0 | 8.72 | -- | N/A | N/A | N/A |
| **Adipic acid** | C6 | 325.0 | 8.75 | Adipic acid-d_4_ | 0.143 | 750 | 0.978 |
| **Pimelic acid** | C7 | 339.0 | 9.45 | Suberic acid-d_4_ | 0.131 | 750 | 0.933 |
| **Suberic acid-d_4_** | C8 | 357.0 | 10.2 | -- | N/A | N/A | N/A |
| **Suberic acid** | C8 | 353.0 | 10.2 | Suberic acid-d_4_ | 0.148 | 750 | 0.987 |
| **Azelaic acid** | C9 | 367.0 | 11.0 | Sebacic acid-d_16_ | 0.145 | 750 | 0.995 |
| **Sebacic acid-d_16_** | C10 | 397.0 | 11.7 | -- | N/A | N/A | N/A |
| **Sebacic acid** | C10 | 381.0 | 11.8 | Sebacic acid-d_16_ | 0.147 | 750 | 0.994 |
