# Supplementary material for: Urine dicarboxylic acids change in pre-symptomatic Alzheimer’s disease and reflect loss of energy capacity and hippocampal volume
Source: PLoS One. 2020 Apr 16;15(4):e0231765. doi: 10.1371/journal.pone.0231765 (PMC7162508; doi:10.1371/journal.pone.0231765)
Supplement: S2 Table — P values < 0.05 are shown in bold italics. (DOCX) [file pone.0231765.s002.docx]

S2 Table. Distribution, proportion, and intergroup comparisons of DCA species normalized for urine volume (ng/mL) between clinical groups. P values < 0.05 are shown in bold italics.

| **Species** | **Classification** | **n** | **Mean ± SD (95% CI)**  **[ng/mL]** | **CV** | **p values** | |
| --- | --- | --- | --- | --- | --- | --- |
| **Malonic acid**  **(C3)** | CH  CH-NAT  CH-PAT  AD | 76  45  31  25 | 177.4 ± 155.8 (141.8 – 213.0)  170.4 ± 111.7 (136.9 – 204)  187.4 ± 205.4 (112.1 – 262.8)  213.8 ± 126.2 (160.5 – 267.1) | 0.878  0.656  1.096  0.590 | CH vs AD  CH-NAT vs CH-PAT  CH-NAT vs AD  CH-PAT vs AD | 0.0603  0.6975  0.1362  0.1092 |
| **Succinic acid**  **(C4)** | CH  CH-NAT  CH-PAT  AD | 76  45  31  25 | 2911 ± 2213 (2406 – 3417)  3074 ± 2398 (2354 – 3795)  2674 ± 1924 (1968 – 3380)  2661 ± 1471 (2040 – 3283) | 0.760  0.780  0.720  0.553 | CH vs AD  CH-NAT vs CH-PAT  CH-NAT vs AD  CH-PAT vs AD | 0.8695  0.6140  0.9950  0.7174 |
| **Glutaric acid**  **(C5)** | CH  CH-NAT  CH-PAT  AD | 76  45  31  25 | 395.9 ± 285.6 (330.6 – 461.1)  398.0 ± 261.3 (319.5 – 476.5)  392.7 ± 322.1 (274.6 – 510.9)  284.3 ± 130.3 (229.3 – 339.3) | 0.723  0.657  0.820  0.458 | CH vs AD  CH-NAT vs CH-PAT  CH-NAT vs AD  CH-PAT vs AD | 0.1938  0.5847  0.1396  0.4635 |
| **Adipic acid**  **(C6)** | CH  CH-NAT  CH-PAT  AD | 76  45  31  25 | 971.3 ± 1187 (700.1 – 1243)  926.1 ± 1192 (568.0 – 1284)  1037 ± 1196 (598.3 – 1476)  1002 ± 689.3 (711.0 – 1293) | 1.222  1.287  1.153  0.688 | CH vs AD  CH-NAT vs CH-PAT  CH-NAT vs AD  CH-PAT vs AD | 0.2356  0.9916  0.2836  0.2992 |
| **Pimelic acid**  **(C7)** | CH  CH-NAT  CH-PAT  AD | 76  45  31  25 | 642.7 ± 437.6 (542.8 – 742.7)  599.4 ± 419.9 (473.2 – 725.5)  705.7 ± 461.7 (536.3 – 875.0)  1032 ± 760.0 (711.5 – 1353) | 0.681  0.701  0.654  0.736 | CH vs AD  CH-NAT vs CH-PAT  CH-NAT vs AD  CH-PAT vs AD | ***0.0033***  0.1484  ***0.0019***  ***0.0474*** |
| **Suberic acid**  **(C8)** | CH  CH-NAT  CH-PAT  AD | 76  45  31  25 | 834.1 ± 567.8 (704.4 – 963.9)  803.8 ± 599.1 (623.8 – 983.8)  878.1 ± 525.6 (685.3 – 1071)  1249 ± 878.4 (878.4 – 1620) | 0.681  0.745  0.599  0.703 | CH vs AD  CH-NAT vs CH-PAT  CH-NAT vs AD  CH-PAT vs AD | ***0.0175***  0.4120  ***0.0156***  0.0850 |
| **Azelaic acid**  **(C9)** | CH  CH-NAT  CH-PAT  AD | 76  45  31  25 | 638.6 ± 685.3 (482 – 795.2)  544.1 ± 549.5 (379.0 – 709.2)  775.8 ± 835.6 (469.3 – 1082)  1256 ± 1290 (711.3 – 1801) | 1.073  1.010  1.077  1.027 | CH vs AD  CH-NAT vs CH-PAT  CH-NAT vs AD  CH-PAT vs AD | ***0.0010***  0.0757  ***0.0003***  ***0.0455*** |
| **Sebacic acid (C10)** | CH  CH-NAT  CH-PAT  AD | 76  45  31  25 | 107.5 ± 131.1 (77.5 – 137.4)  108.8 ± 156.6 (61.76 – 155.8)  105.5 ± 83.73 (74.79 – 136.2)  155.9 ± 161.8 (87.61 – 224.3) | 1.220  1.439  0.794  1.038 | CH vs AD  CH-NAT vs CH-PAT  CH-NAT vs AD  CH-PAT vs AD | ***0.0051***  0.2381  ***0.0010***  0.1431 |
| **Sum C3-C10** | CH  CH-NAT  CH-PAT  AD | 76  45  31  25 | 6679 ± 3920 (5783 – 7574)  6625 ± 3982 (5429 – 7821)  6756 ± 3894 (5328 – 8185)  7855 ± 4539 (5939 – 9772) | 0.587  0.601  0.576  0.578 | CH vs AD  CH-NAT vs CH-PAT  CH-NAT vs AD  CH-PAT vs AD | 0.2230  0.8336  0.2258  0.3579 |
