# Supplementary material for: Urine dicarboxylic acids change in pre-symptomatic Alzheimer’s disease and reflect loss of energy capacity and hippocampal volume
Source: PLoS One. 2020 Apr 16;15(4):e0231765. doi: 10.1371/journal.pone.0231765 (PMC7162508; doi:10.1371/journal.pone.0231765)
Supplement: S3 Table — Carbon number (C3-C10), negative ion (m/z), retention time (RT), deuterated internal standards, detection linear range, and correlation (R2). (DOCX) [file pone.0231765.s003.docx]

S3 Table. Percent distribution, proportion, and intergroup comparison of DCA species between clinical and biochemical groups. P values < 0.05 are shown in bold italics.

| **Species** | **Classification** | **n** | **% Mean ± SD (95% CI)** | **CV** | **p values** | |
| --- | --- | --- | --- | --- | --- | --- |
| **Malonic acid**  **(C3)** | CH  CH-NAT  CH-PAT  AD | 76  45  31  25 | 2.945 ± 1.701 (2.556 – 3.334)  2.988 ± 1.586 (2.511 – 3.464)  2.833 ± 1.881 (2.193 – 3.573)  2.904 ± 1.268 (2.368 – 3.439) | 0.578  0.531  0.652  0.437 | CH vs AD  CH-NAT vs CH-PAT  CH-NAT vs AD  CH-PAT vs AD | 0.6389  0.4944  0.9551  0.3669 |
| **Succinic acid**  **(C4)** | CH  CH-NAT  CH-PAT  AD | 76  45  31  25 | 41.86 ± 12.13 (39.09 – 44.64)  44.12 ± 11.85 (40.56 – 47.68)  38.59 ± 11.96 (34.21 – 42.98)  34.72 ± 10.59 (30.24 – 39.19) | 0.290  0.269  0.310  0.305 | CH vs AD  CH-NAT vs CH-PAT  CH-NAT vs AD  CH-PAT vs AD | ***0.0113***  0.0869  ***0.0027***  0.1959 |
| **Glutaric acid**  **(C5)** | CH  CH-NAT  CH-PAT  AD | 76  45  31  25 | 6.346 ± 3.387 (5.572 – 7.120)  6.582 ± 3.528 (5.522 – 7.641)  6.004 ± 3.198 (4.831 – 7.178)  4.353 ± 2.251 (3.420 – 5.303) | 0.534  0.536  0.533  0.517 | CH vs AD  CH-NAT vs CH-PAT  CH-NAT vs AD  CH-PAT vs AD | ***0.0087***  0.4490  ***0.0066***  0.0653 |
| **Adipic acid**  **(C6)** | CH  CH-NAT  CH-PAT  AD | 76  45  31  25 | 13.76 ± 9.397 (11.61 – 15.91)  13.76 ± 9.584 (10.88 – 16.63)  13.76 ± 9.276 (10.36 – 17.16)  13.10 ± 4.718 (11.11 – 15.09) | 0.683  0.697  0.674  0.360 | CH vs AD  CH-NAT vs CH-PAT  CH-NAT vs AD  CH-PAT vs AD | 0.4636  0.9916  0.5049  0.5275 |
| **Pimelic acid**  **(C7)** | CH  CH-NAT  CH-PAT  AD | 76  45  31  25 | 10.26 ± 3.793 (9.398 – 11.13)  9.749 ± 3.917 (8.573 – 10.93)  11.01 ± 3.533 (9.716 – 12.31)  12.72 ± 2.912 (11.49 – 13.95) | 0.370  0.402  0.321  0.229 | CH vs AD  CH-NAT vs CH-PAT  CH-NAT vs AD  CH-PAT vs AD | ***0.0035***  0.2609  ***0.0032***  ***0.0320*** |
| **Suberic acid**  **(C8)** | CH  CH-NAT  CH-PAT  AD | 76  45  31  25 | 13.25 ± 5.284 (12.04 – 14.46)  12.60 ± 4.788 (11.16 – 14.04)  14.20 ± 5.885 (12.04 – 16.35)  15.61 ± 3.642 (14.08 – 17.17) | 0.400  0.380  0.415  0.233 | CH vs AD  CH-NAT vs CH-PAT  CH-NAT vs AD  CH-PAT vs AD | ***0.0161***  0.3329  ***0.0083***  0.1339 |
| **Azelaic acid**  **(C9)** | CH  CH-NAT  CH-PAT  AD | 76  45  31  25 | 9.784 ± 6.603 (8.275 – 11.29)  8.475 ± 5.203 (6.912 – 10.04)  11.68 ± 7.937 (8.772 – 14.59)  14.43 ± 7.770 (11.14 – 17.71) | 0.675  0.614  0.679  0.539 | CH vs AD  CH-NAT vs CH-PAT  CH-NAT vs AD  CH-PAT vs AD | ***0.0022***  0.0689  ***0.0002***  0.1385 |
| **Sebacic acid (C10)** | CH  CH-NAT  CH-PAT  AD | 76  45  31  25 | 1.788 ± 1.712 (1.396 – 2.179)  1.734 ± 1.922 (1.157 – 2.311)  1.865 ± 1.378 (1.360 – 2.370)  2.163 ± 1.763 (1.418 – 2.907) | 0.958  1.109  0.739  0.815 | CH vs AD  CH-NAT vs CH-PAT  CH-NAT vs AD  CH-PAT vs AD | 0.0721  0.1806  ***0.0158***  0.5871 |
| **Sum C4 + C5** | CH  CH-NAT  CH-PAT  AD | 76  45  31  25 | 48.21 ± 13.05 (45.23 – 51.19)  50.70 ± 12.37 (46.98 – 54.42)  44.60 ± 13.35 (39.70 – 49.50)  39.07 ± 11.14 (34.37 – 43.77) | 0.271  0.244  0.299  0.285 | CH vs AD  CH-NAT vs CH-PAT  CH-NAT vs AD  CH-PAT vs AD | ***0.0059***  0.0722  ***0.0011***  0.1576 |
| **Sum C7 – C10** | CH  CH-NAT  CH-PAT  AD | 76  45  31  25 | 35.09 ± 12.14 (32.31 – 37.86)  32.56 ± 10.86 (29.30 – 35.82)  38.76 ± 13.12 (33.94 – 43.57)  44.92 ± 9.783 (40.79 – 49.06) | 0.346  0.334  0.339  0.218 | CH vs AD  CH-NAT vs CH-PAT  CH-NAT vs AD  CH-PAT vs AD | ***0.0004***  ***0.0368***  ***<0.0001***  0.0604 |
