# Supplementary material for: Urine dicarboxylic acids change in pre-symptomatic Alzheimer’s disease and reflect loss of energy capacity and hippocampal volume
Source: PLoS One. 2020 Apr 16;15(4):e0231765. doi: 10.1371/journal.pone.0231765 (PMC7162508; doi:10.1371/journal.pone.0231765)
Supplement: S1 Fig — (DOCX) [file pone.0231765.s004.docx]

**S1 Fig. GC MS method.**
