# Supplementary material for: Urine dicarboxylic acids change in pre-symptomatic Alzheimer’s disease and reflect loss of energy capacity and hippocampal volume
Source: PLoS One. 2020 Apr 16;15(4):e0231765. doi: 10.1371/journal.pone.0231765 (PMC7162508; doi:10.1371/journal.pone.0231765)
Supplement: Attachment — Submitted filename: Responses to reviewers.docx [file pone.0231765.s005.docx]

**Responses to the reviewers**

**Reviewer 1**

*But would like to suggest an open search for lipid metabolites as urinary biomarkers in the future.* We add a statement to recommend an open search for lipid metabolites in our “limitations” paragraph.

*But there is a theory about urinary biomarker which will support the manuscript but not mentioned. The theory is in the paper with recent progress of urinary protein biomarkers on early AD animal model (J Alzheimers Dis. (2018) 66:613–637. doi: 10.3233/JAD-180412).* We add the recommended reference.

**Reviewer 2**

1. *Please give the precise structure of C4-C5 DCAs and C7-C10 DCAs, are they oxidized?* We have added the structure of the DCA’s to the Supp figure 1. We have also included more GCMS method details with the structure of the PFBBr species that we detect in the method; this is interesting as only one PFBBr adduct is detected.
2. *Single ion monitoring (SIM) mode based on triple quad mass spectrometry is a Quantitative platform for targeted analysis, but is not suitable for the finding of biomarkers. Therefore, although the authors targeted dicarboxylic acids because they are implicated in several processes associated with AD pathology, I guess readers may also need to explain why and how the C4-C5 DCAs and C7-C10 DCAs were selected for SIM analysis. Specifically, whether some other DCAs such as smaller than C4 or bigger than C 10 were detected in urine, or there is no statistical differences for these DCAS between AD and cognitively healthy groups.* The recovery yield for C3 and DCAs greater than 10 turned out to be poor and we were not able to optimize the method within these experiments. The values for C6, and C10 were of good quality but they were not significantly different between the groups. We concentrated on those DCAs that we found to be different between CH and AD and which yielded good quality data. We have started to look at larger DCA’s with a new approach, but this is beyond the scope of this manuscript. We used a triple quad GCMS and used the SIM approach because the DCAs all fell apart under conditions we tried. As we mention in the limitations section, we state clearly that a simpler assay will be needed for widespread use, but we hope that these initial results that we report will stimulate efforts to look further at urine as a biomarker with potential for screening of pre-symptomatic AD.
3. *However, some details of the analysis method may be ignored. For example, gas chromatographic methods coupled with mass spectrometry is widely used for the detection of dicarboxylic acids, but the efficiency and stability of reaction products and several derivatization agents may be different, so the detection limit (LOD) and relative standard deviation (RSD) of C4,C5… C10 should be given, 0therwise, the relationship between signal intensity and content cannot be accurately judged.* In our original submission, we had included the LOD, dynamic ranges, 95% CI, means (SD) for all values of DCAs, and the R^2^ of internal standards in Tables 1-3. Perhaps the reviewer did not have access to this supplementary data; these parameters give clear description of the data quality.
4. *In Fig 2(B). Changes in urine DCA in clinical groups, if I am not wrong, there are 25 black triangles, which is not inconsistent with 24 AD samples.* Good catch! We had stated we had an N of 24 for AD in some texts, Tables and in scatter plots in figure 2B; **it is 25**! We had 25 correctly in other parts. We have corrected this mistake throughout, as indicated in the track changes. Thanks!
